# Exhalation metabolomics: A new force in revealing the impact of ozone pollution on respiratory health

**DOI:** 10.1016/j.eehl.2024.05.001

**Published:** 2024-05-09

**Authors:** Chen Tao, Peter Mettke, Yaru Wang, Xue Li, Ligang Hu

**Affiliations:** a State Key Laboratory of Environmental Chemistry and Ecotoxicology, Research Center for Eco-Environmental Sciences, Chinese Academy of Sciences, Beijing 100085, China; b Taishan Institute for Ecology and Environment, Jinan 250100, China; c Atmospheric Chemistry Department, Leibniz Institute for Tropospheric Research, Leipzig 04318, Germany; d Institute of Mass Spectrometry and Atmospheric Environment, Guangdong Provincial Key Laboratory of Speed Capability Research, Jinan University, Guangzhou 510632, China; e School of Environment, Hangzhou Institute for Advanced Study, University of Chinese Academy of Sciences, Hangzhou 310000, China; f School of Environment and Health, Jianghan University, Wuhan 430056, China

## Abstract

• Near-surface ozone pollution has a significant impact on respiratory health.
• Lung microenvironment is involved in respiratory health effects of ozone pollution.
• Exhalation metabolomics provides a new method to explore the respiratory health effects of ozone pollution.
• Exhalation metabolomics could be a potential basis for concentration limits in ozone pollution control.

## Graphical abstract

**Figure:**
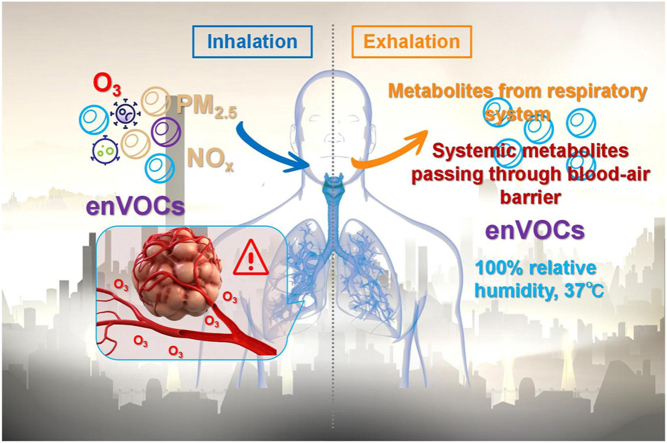

## 1 Near-surface ozone

Ozone (O_3_) is mainly distributed in the stratosphere of the atmosphere, with relatively low concentrations near the ground, but it has profound implications for climate change, ecological environment, and human health. Research on the spatial and temporal variation characteristics of near-ground O_3_ and its precursors, as well as qualitative correlation analysis and model simulation on short-term scales, have shown that the concentration of near-ground O_3_ has strong seasonal variations and spatial agglomeration characteristics [1]. From the perspective of the temporal variation of near-surface O_3_ and its precursor concentrations, in the winter half year, the precursor accumulation is not conducive to the formation of O_3_ through photochemical reactions due to low temperatures and weak radiation, while the opposite is true in the summer half year, especially when the sunshine duration is long, the radiation is strong, the temperature is high, and photochemical reactions are active. Meteorological conditions are conducive to the conversion of nitrogen oxides (NO_x_) and volatile organic compounds (VOCs) and other precursors of O_3_ into secondary products O_3_ through photochemical reactions [2]. In addition to meteorological conditions, there are many factors that affect near-surface O_3_ levels, including geographical location, atmospheric diffusion, and local source emissions, which jointly affect the relative proportion and abundance of O_3_ precursors such as NO_x_ and VOCs [3]. The increase in near-ground O_3_ content is mainly due to human activities, such as vehicle exhaust from transportation, waste gas from petrochemical production, coal-fired power generation, and other biomass combustion, which emit VOCs and NO_x_ into the atmosphere.

## 2 Ozone pollution

Near-surface O_3_, as a global pollutant, is one of the research hotspots in the field of environmental science. Since the 1990s, due to strict emission control, severe O_3_ pollution in many cities and regions in Europe and America has been alleviated. However, in recent decades, developing countries have experienced rapid industrialization and economic growth, resulting in a significant increase in anthropogenic O_3_ precursor emissions [4]. So far, O_3_ pollution has gradually gained deeper understanding and attention from people. For example, the United States Environmental Protection Agency (EPA) and National Ambient Air Quality Standards (NAAQS) regulations, the European Union (EU) Air Quality Directive regulations, and China's atmospheric environmental quality standards have all clearly provided limits for air O_3_ concentration. The monitoring in accordance with the air quality standards reveals that O_3_ pollution has increasingly received attention in industrial countries or regions and has become another major air pollutant after fine particulate matter (PM_2.5_). With the improvement of the atmospheric environment governance system and the adjustment of energy structure, the seasonal composition of pollutants has undergone significant changes, and the PM_2.5_ content in the atmosphere has been effectively controlled. However, at the same time, the concentration of O_3_ near the ground has significantly increased, even becoming the primary pollutant in many cities during summer [5]. High concentrations of O_3_, as a characteristic product of secondary pollution, are particularly prominent in densely populated and rapidly urbanized areas. For example, observational studies around the Beijing-Tianjin-Hebei, Yangtze River Delta, and Pearl River Delta urban agglomerations in China have shown that with climate change and intensified pollution emissions, O_3_ concentrations and exceeding standards in major urban agglomerations are increasing year by year [6]. Meanwhile, according to *the 2019 Report on the State of the Ecology and Environment in China*, O_3_ is the only air pollutant that has maintained an upward trend in concentration in the past five years. In addition, based on the migration of new manufacturing capacity to some developing countries in Southeast Asia, Central Asia, and Africa, the hidden concerns of O_3_ pollution in these regions in the future are faintly visible. From this, it can be seen that O_3_ pollution has become a wide-ranging environmental problem that urgently needs to be addressed at present.

## 3 Elemental oxygen and respiratory health

Elemental oxygen is crucial for the survival of oxygen-consuming organisms and plays an important role in the physiological processes of metabolism. High-concentration oxygen inhalation is widely used in the treatment of acute and chronic hypoxemia. However, excessive inhalation of high levels of oxygen can lead to fatal respiratory injury in both humans and animals. The biological damage of high levels of oxygen to the lungs has been fully confirmed [7], and this effect has been shown to be universal in mammals. However, the mechanism of respiratory injury caused by high levels of oxygen inhalation is not fully understood [8], and even less is known about the mechanism of respiratory health effects caused by O_3_ exposure. High concentrations of O_3_ near the ground are more harmful than traditional high levels of oxygen inhalation, and the health problems it causes are common in different countries and regions, with a growing trend in population-weighted O_3_ concentrations. According to relevant research estimates, the global death toll caused by O_3_ exposure in 2019 reached 472,000 people [9]. Large-scale population survey shows that both short-term and long-term exposure to O_3_ increases the mortality caused by non-accidental factors [10], and is related to the deterioration of human cardiopulmonary function and the incidence rate of respiratory diseases [11]. Meanwhile, O_3_ inhalation exposure can cause eosinophilic airway inflammation, increasing susceptibility or allergenicity to respiratory pathogens or allergens [12]. Studies on humans and experimental animals have shown that inhaling O_3_ can exacerbate hyperresponsiveness and immune and inflammatory responses of asthma-type airways. In addition, an increase in O_3_ exposure concentration is associated with a decrease in respiratory function such as forced expiratory volume (FEV) and forced vital capacity (FVC) in the first second [13].

## 4 Exploring the respiratory microenvironment and its significance

The latest advances in non-cultivation techniques for microorganisms indicate that microorganisms exist even in the lungs of healthy individuals [14]. Microorganisms widely present in the air are inhaled into the lungs through the mouth and nose, and lung microorganisms maintain a dynamic balance between self-migration, colonization, and host clearance. After lung injury, the homeostasis of lung micro-environment factors is disrupted. Due to the formation of new selection pressures on lung microbiota by the respiratory micro-environment, changes in the structure of lung microbiota communities are often manifested as an increase in the proportion of competitive lung microbiota in the entire community, as well as a relative decrease or even extinction of competitive lung microbiota [15]. A study has found that microorganisms in healthy lungs seem to be more affected by microbial transplantation and elimination than the associated respiratory micro-environment survival conditions, but this pattern may be reversed by changes in the respiratory micro-environment during the development of lung diseases. Therefore, changes in the composition of lung microbiota are usually associated with lung injury [16]. In bronchiectasis, current research suggests that changes in the composition of lung microbiota caused by events such as antibiotic treatment and inflammatory response may have an impact on downstream immune system function, leading to chronic infections caused by microbial pathogens [17]. *Pseudomonas*, *Streptococcus*, and *Achromobacterium* can be detected in the lungs of adult patients with pulmonary cystic fibrosis. *Propionibacterium* is also present in high abundance in the lungs of both adult and child patients with pulmonary cystic fibrosis. Chronic obstructive pulmonary disease (COPD) is highly correlated with the genera *Streptococcus* and *Mycobacterium*, and there is a decrease in pulmonary microbial diversity. The relative abundance of *Haemophilus*, *Streptococcus*, *Neisseria*, and *Vibrio* genera in the lungs of asthma patients is relatively high. There is also a relatively high abundance of *Staphylococcus* species in the lungs of patients with cystic fibrosis and children with asthma. The detection of lung microbial diversity under specific pathological conditions through clinical samples of lung diseases is an important entry point and theoretical basis for understanding the relationship between respiratory microenvironment and respiratory injury. Meanwhile, due to the typicality and specificity of certain pulmonary microorganisms in lung diseases, they can also be used to distinguish and evaluate lung injury [18].

## 5 Utilizing exhalation metabolomics to assess environmental and health effects

The research objects of metabolomics are endogenous metabolites and exogenous substances ingested into the body. By analyzing the changes of these metabolites in body fluids and tissues, researchers can assess the impact of internal and external factors like gene expression and protein regulation on the body's state at the overall biological level. Any physiological, pathological, or other changes in the body will affect the concentration of metabolites or cause changes in metabolic flow. According to the source of the metabolite sample, the metabolome can be divided into blood, urine, exhalation, etc. Metabolomics can be used to reflect the health effects of environmental pollutants on living organisms, such as by studying the effects of PM_2.5_ in the air on the health of patients with COPD through urine metabolomics [19], or the effects of air pollution on the health of normal individuals through blood metabolomics [20]. If VOCs in the atmospheric environment are referred to as exogenous VOCs, the corresponding VOCs from metabolic processes in the body are referred to as endogenous VOCs. Changes in the concentration and composition of endogenous VOCs in human exhalation are related to health or disease [21]. Exhalation is a type of biological sample that has received much attention recently. It contains various endogenous and exogenous substances, which can reflect the health status of the organism as well as the intake, metabolism, and accumulation of exogenous substances. At present, exhalation metabolomics technology has been widely used for the detection and diagnosis of VOC components in exhaled air across different physiological or pathological states of the human body, as well as the identification and analysis of potential metabolic markers [[22], [23], [24]]. In addition, exhalation metabolomics can be used to reflect the impact of environmental pollutants on *in vivo* endogenous VOCs, such as evaluating the impact of offshore oil spills on the health of wild whales through exhalation metabolomics [25] or the impact of traffic-related air pollution on the respiratory system [26]. Studies *in vitro* have shown that there are differences in the endogenous VOC fingerprint of lung epithelial cell inflammation caused by oxidative factors such as hydrogen peroxide and other biological factors [27], indicating that the endogenous VOC changes caused by O_3_ inhalation exposure may also be specific.

## 6 Investigating the respiratory microenvironmental response to ozone inhalation exposure

The tolerance of microorganisms colonized in the lungs to oxidative stress varies greatly, and the lung microbiota of respiratory failure patients receiving high-concentration oxygen inhalation treatment has undergone significant changes [28]. Based on oxidative differences, O_3_ exposure may have a greater impact on lung microbiota compared to high level of oxygen inhalation. Although high oxygen levels in the patient's airway and extra-alveolar environment are not common, oxidative stress is a key determinant of bacterial community structure [29]. The tolerance of lung-associated bacteria to oxidative stress varies greatly, ranging from specialized anaerobic bacteria (such as *Prevotella*) and facultative anaerobic bacteria (such as *Pseudomonas aeruginosa*) to specialized aerobic bacteria (such as *Mycobacterium tuberculosis*). *Staphylococcus aureus* is the most common pathogen of ventilator-associated pneumonia and has evolved many mechanisms to tolerate oxidative stress. Due to the key characteristic of innate immune response being the production of reactive oxygen species by inflammatory cells in the immune system, pulmonary inflammation is characterized by high oxidative stress. Therefore, there may be overlapping metabolic mechanisms in the adaptation strategies of respiratory pathogens, allowing them to survive through metabolic collaboration in innate immune responses and environments with high oxygen levels [30]. Acute high-level oxygen can alter the bacterial community composition of mouse lung microbiota, selectively promoting the enrichment of oxygen-tolerant groups in the lungs (such as *Staphylococcus*). Hyperoxia-induced pulmonary microbiota dysbiosis occurs before lung injury. In mice with the same gene and exposure to high levels of oxygen for the same duration, changes in pulmonary microbiota are associated with changes in pulmonary inflammation. Sterile mice are less affected by high-oxygen-induced lung injury, and systemic antibiotic therapy selectively reduces the susceptibility of conventionally fed mice to such injury [31]. High-level oxygen conditions alter lung oxidative capacity and can affect lung microbiota, while high levels of oxygen induction and destruction of lung microbiota are associated with lung injury. It can be inferred that O_3_ induced disruption of lung microbiota is a potential factor affecting respiratory health.

## 7 Integrating natural inhalation exposure with exhalation imprinting: A necessity

Exhalation metabolomics has the characteristics of continuous, non-invasive, and convenient sampling in many metabolome studies [[32], [33], [34]]. Substances directly produced in the blood and crossing through the blood-air barrier or respiratory tract metabolism mix with air to form aerosols, including endogenous VOCs, non-volatile organic compounds embedded in the exhaled breath particles, inorganic gases, etc. Among them, endogenous VOCs are the most studied substances in exhalation metabolomics. When comparing the differences in sampling methods determined by sample sources in metabolomics studies, exhalation metabolomics studies have the advantage of virtually limitless supply available and continuous sampling in a short time frame of seconds. Based on the semi-permeability of the biological blood-air barrier, the observable liquid–gas exchange area during respiration, water vapor evaporation, and the aerodynamic atomization of the liquid film on the inner surface of the lower respiratory tract (bronchioles, respiratory bronchioles, alveolar tubes, etc.) [35], the exhaled metabolites not only reflect the metabolism of related tissues mainly in the lungs, but also reflects secondary and systemic metabolism by presenting metabolites in the blood. Exhalation imprint refers to the sampling and detection process in the study of exhalation metabolomics. In the current breath imprinting technology, the collection methods for breath samples mainly include solid-phase microextraction, gas bag combined with solid-phase microextraction, and online collection. The detection methods for breath samples are mainly divided into gas chromatography-mass spectrometry (GC–MS), liquid chromatography-mass spectrometry (LC–MS), electronic olfaction based on specific sensor arrays, and online mass spectrometry [36]. Among them, online mass spectrometry enables real-time monitoring and continuous analysis of exhaled breath. Online mass spectrometry techniques that have been widely used include proton transfer reaction mass spectrometry (PTR–MS), selective ion flow tube mass spectrometry (SIFT–MS), and secondary electrospray ionization mass spectrometry (SESI–MS). In traditional exhalation metabolomics and other metabolomics studies, detection of metabolism at time points after inhalation exposure has been achieved; however, there is currently a lack of real-time integration technology between inhalation exposure and exhalation imprints. In research fields such as biology, medicine, pharmacy, and environmental science, the real-time response of exhalation metabolomics changes to inhalation exposure has practical significance and application prospects in revealing the immediate health effects of exogenous substances entering the organism through inhalation pathways [37]. At present, in the study of exhalation metabolomics combining inhalation exposure and exhalation imprinting, tracheal infusion or aerosol intratracheal one-time administration is usually used [38], which deviates from the normal physiological state of inhalation exposure. Therefore, there is a practical need to combine natural inhalation exposure with exhalation imprinting technology in real time.

## 8 Outlook

To sum up, O_3_, as a major air pollutant with an obvious concentration rise trend in a wide range, is positively related to the incidence rate and mortality of respiratory diseases (Supplementary Material). Monitoring O_3_ concentrations in exposed environments is relevant to the study of O_3_ health effects. A recent study showed that 98% of COPD deaths could be avoided by reducing O_3_ to the WHO Air Quality Guidelines (AQG) 2021 level. Indoor O_3_ sources contribute less than 5% of COPD deaths. Population growth and aging increase the urgency of O_3_ control [39]. Atmospheric O_3_ concentrations vary during different seasons and at different times of the day. Also, O_3_ concentrations tend to vary with exposure scenarios. Outdoor O_3_ living exposure concentrations could be referenced to regional real-time atmospheric monitoring data. Depending on the precision of the specific study, gridded online monitoring locations may be required when monitoring data are insufficient. O_3_ concentration monitoring for indoor and outdoor occupational exposures, as well as indoor domestic exposures, may require the use of easily deployable point monitoring equipment. In mechanistic studies of O_3_ health effects related to experimental animals, O_3_ exposure concentrations may be set based on relevant air quality standards or experimental purposes.

Exhalation metabolomics may have a positive impact on ozone-related health epidemiological and pathological studies. Exhalation metabolomics provides new tools for epidemiological studies as a non-invasive assay that facilitates large-scale application in epidemiological studies. By collecting and analyzing breath samples from a large number of people, epidemiological researchers may be able to more accurately assess the relationship between O_3_ exposure and the risk of disease, and provide a scientific basis for the formulation of relevant policies and preventive measures. Exhalation metabolomics may facilitate pathological studies by providing new clues and evidence. By analyzing the metabolites in exhaled breath, pathological researchers could understand how O_3_ affects the cells and tissues of the human body, and thus reveal the pathological mechanisms of ozone-induced diseases. Through exhalation metabolomics, people may be able to detect changes in the metabolic state of the human body earlier after O_3_ exposure, thus enabling early diagnosis of related diseases and improving the sensitivity of disease diagnosis, which will help to take timely interventions to prevent further progression of the diseases. Meanwhile, ethical considerations need to be fully taken into account in exhalation metabolomics studies to ensure compliance and morality of the study. This includes protecting the privacy and right to information of clinical volunteers, safeguarding the welfare of laboratory animals, and adhering to ethical norms for sample collection and handling.

Individual differences in samples are a major source of variability in metabolic profiles. The differences in diet, lifestyle, underlying disease, and others can directly affect the metabolite composition in exhalation. For example, different dietary habits may lead to significant changes in the concentration of certain metabolites, which in turn affects the results of metabolic profiling. In addition, differences in lifestyle, such as the amount of exercise and sleeping habits, may also have an impact on the metabolic profile. Therefore, how to effectively control these interfering factors to obtain more accurate and consistent metabolic profiling data is a key issue to be addressed in the application of exhalation metabolomics. The lack of standardized protocols is another important challenge for exhalation metabolomics. Since a well-established standardized process has not yet been established, there may be significant differences in sample collection, processing, and analysis between different laboratories or research institutes, which directly leads to the problems of incomparability and reproducibility of research results. In order to solve this problem, there is a need to establish a unified standardized protocol for exhalation metabolomics, including standard operating procedures for sample collection, uniform methods for data preprocessing and analysis, and standard specifications for results interpretation.

**Fig. 1:**
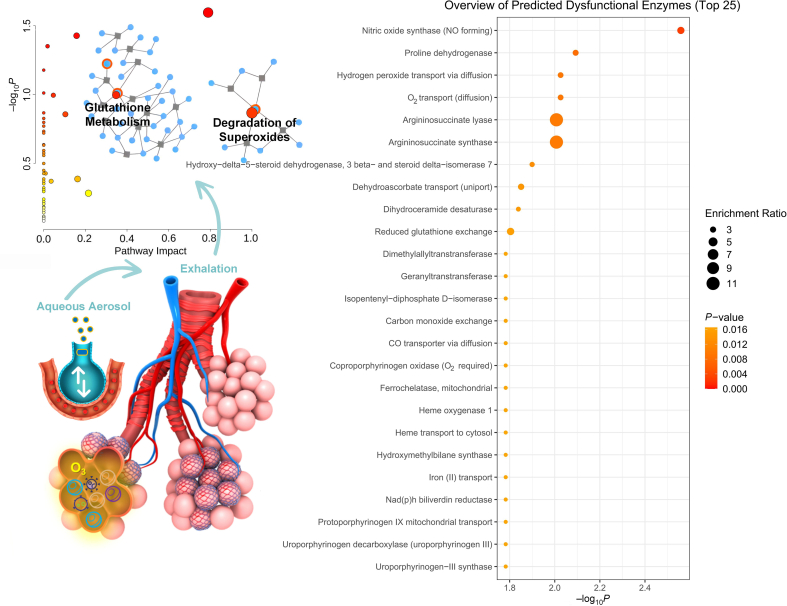
The lung is the organ with the largest unfolded area of communication with the outside environment and is the site of direct action of inhaled pollutants, including O_3_. Text mining of selected literature related to respiratory diseases (HMDB, PubMed) was used to explore the perturbations of O_3_ inhalation exposure on human metabolic streams, which include superoxide degradation and glutathione metabolism that may be accompanied by a range of enzyme dysfunctions.

The health effects of O_3_ inhalation exposure on the airway mainly include inducing oxidative stress and airway inflammation, but there is still a lack of deeper mechanism research (Fig. 1). The consequences of O_3_ inhalation exposure leading to respiratory injury include both physiological and pathological changes in the lung tissue itself, as well as changes in the lung microbiota. The direct oxidative stress of O_3_ on lung microbiota and the indirect killing of lung microbiota by tissue inflammatory response after lung injury may be the main drive for the remodeling of lung microbiota. The changes in lung microbiota under O_3_ stress exacerbate the pulmonary sensitivity to O_3_, and the underlying mechanisms are not yet clear. Meanwhile, the metabolic dominant communities and their pathogenicity and pro-inflammatory effects in lung microbiota under O_3_ stress are still unclear. By integrating exhalation metabolomics and other physiological and biochemical indicators, the mechanism of O_3_ effect on respiratory health would be revealed, providing a more substantial scientific basis for objectively evaluating the rationality of O_3_ limits in existing air quality standards. Meanwhile, the exhalation metabolomics mentioned in this article has the potential to become an effective tool for future environmental exposure science, as it could also be applied to other environmental pollutants or artificial chemicals.

## CRediT authorship contribution statement

**Chen Tao:** Conceptualization, Investigation, Visualization, Writing – original draft, Writing – review & editing. **Peter Mettke:** Investigation, Writing – review & editing. **Yaru Wang:** Investigation, Writing – review & editing. **Xue Li:** Investigation, Writing – review & editing. **Ligang Hu:** Funding acquisition, Supervision, Writing – review & editing.

## Declaration of competing interests

The authors have declared no conflicts of interest.
