# Supplementary material for: Exhalation metabolomics: A new force in revealing the impact of ozone pollution on respiratory health
Source: Eco Environ Health. 2024 May 9;3(4):407–11. doi: 10.1016/j.eehl.2024.05.001 (PMC11541422; doi:10.1016/j.eehl.2024.05.001)
Supplement: Multimedia component 1 [file mmc1.docx]

***Supplementary Material***

***for***

**Exhalation metabolomics: A new force in revealing the impact of ozone pollution on respiratory health**

Chen Tao^1,2^, Peter Mettke^3^, Yaru Wang^3^, Xue Li^4^, Ligang Hu^1,2,5,6*^

^1^State Key Laboratory of Environmental Chemistry and Ecotoxicology, Research Center for Eco-Environmental Sciences, Chinese Academy of Sciences, Beijing 100085, China

^2^Taishan Institute for Ecology and Environment, Jinan 250100, China

^3^Atmospheric Chemistry Department, Leibniz Institute for Tropospheric Research, Leipzig 04318, Germany

^4^Institute of Mass Spectrometry and Atmospheric Environment; Guangdong Provincial Key Laboratory of Speed Capability Research, Jinan University, Guangzhou 510632, China

^5^School of Environment, Hangzhou Institute for Advanced Study, University of Chinese Academy of Sciences, Hangzhou 310000, China

^6^School of Environment and Health, Jianghan University, Wuhan 430056, China

*Corresponding Author

Ligang Hu (lghu@rcees.ac.cn)

**Content**

Figure: 1

Table: 1

References: 38


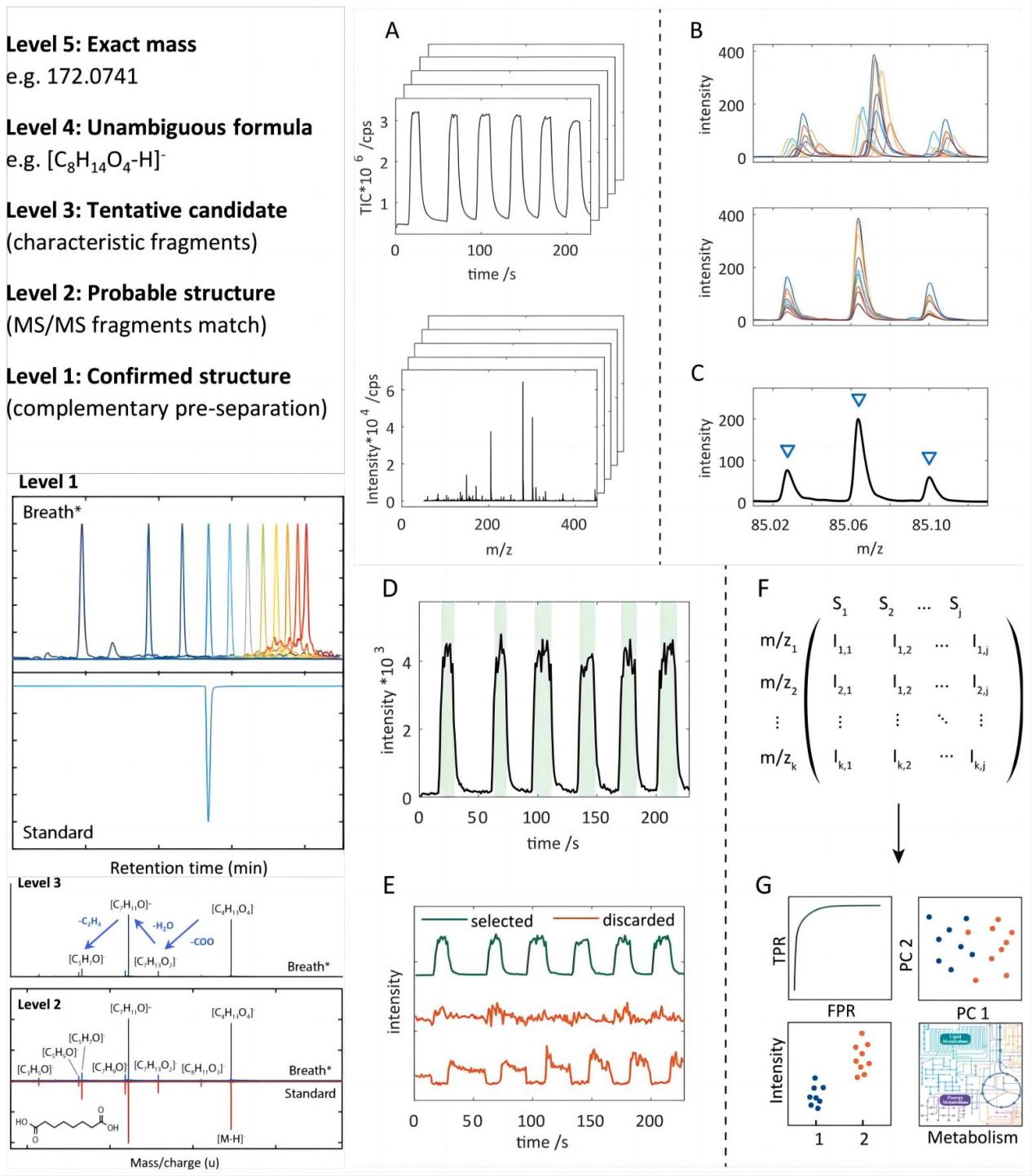


Figure S1. Research flowchart in exhalation metabolomics [1]. Levels of compound identification based on the approach [2], the example flowcharts shown here are from exhaled breath condensate analysis and on-line exhaled breath analysis with HRMS/MS. (A) Conversion of the raw data into an open file format (e.g., mzXML or mzML). (B) Alignment of the spectra using reference peaks. (C) Peak-picking using the average spectrum of all measurements. (D) Generation of time-traces and detection of time points of interest (green). (E) Feature reduction to compounds of interest. (F) Generation of the final intensity matrix by averaging the intensities during the exhalations. (G) Subjection of the final matrix to the downstream statistical analysis.

Table S1. Prospective exhaled metabolites relevant to ozone respiratory effects identified by text mining (HMDB, PubMed).

| **Metabolite** | **HMDB** | **KEGG** | **Chemical formula** | **Reference** |
| --- | --- | --- | --- | --- |
| Acetate | HMDB0000042 | C00033 | C_2_H_4_O_2_ | [3] |
| Adenosine | HMDB0000050 | C00212 | C_10_H_13_N_5_O_4_ | [4] |
| Ammonia | HMDB0000051 | C00014 | H_3_N | [5] |
| D-Glucose | HMDB0000122 | C00221 | C_6_H_12_O_6_ | [6] |
| Glutathione | HMDB0000125 | C00051 | C_10_H_17_N_3_O_6_S | [7] |
| Formate | HMDB0000142 | C00058 | CH_2_O_2_ | [8] |
| Palmitic acid | HMDB0000220 | C00249 | C_16_H_32_O_2_ | [9] |
| Quinolinic acid | HMDB0000232 | C03722 | C_7_H_5_NO_4_ | [10] |
| Propanoate | HMDB0000237 | C00163 | C_3_H_6_O_2_ | [3] |
| Uric acid | HMDB0000289 | C00366 | C_5_H_4_N_4_O_3_ | [11] |
| Urea | HMDB0000294 | C00086 | CH_4_N_2_O | [12] |
| Chloride | HMDB0000492 | C00698 | Cl | [13] |
| Potassium | HMDB0000586 | C00238 | K | [14] |
| Sodium | HMDB0000588 | C01330 | Na | [14] |
| Copper | HMDB0000657 | C00070 | Cu | [15] |
| Kynurenine | HMDB0000684 | C00328 | C_10_H_12_N_2_O_3_ | [10] |
| Isopropanol | HMDB0000863 | C01845 | C_3_H_8_O | [16] |
| Leukotriene B4 | HMDB0001085 | C02165 | C_20_H_32_O_4_ | [17] |
| Anthranilic acid | HMDB0001123 | C00108 | C_7_H_7_NO_2_ | [10] |
| Prostaglandin F2 | HMDB0001139 | C00639 | C_20_H_34_O_5_ | [18] |
| Leukotriene C4 | HMDB0001198 | C02166 | C_30_H_47_N_3_O_9_S | [19] |
| Prostaglandin E2 | HMDB0001220 | C00584 | C_20_H_32_O_5_ | [19] |
| Prostaglandin D2 | HMDB0001403 | C00696 | C_20_H_32_O_5_ | [20] |
| Prostaglandin F2b | HMDB0001483 | C02314 | C_20_H_34_O_5_ | [18] |
| Asymmetric dimethylarginine | HMDB0001539 | C03626 | C_8_H_18_N_4_O_2_ | [21] |
| Acetone | HMDB0001659 | C00207 | C_3_H_6_O | [16] |
| Isobutyric acid | HMDB0001873 | C02632 | C_4_H_8_O_2_ | [3] |
| Methanol | HMDB0001875 | C00132 | CH_4_O | [3] |
| 3-Nitrotyrosine | HMDB0001904 | NA | C_9_H_10_N_2_O_5_ | [22] |
| Docosahexaenoic acid | HMDB0002183 | C06429 | C_22_H_32_O_2_ | [23] |
| Leukotriene E4 | HMDB0002200 | C05952 | C_23_H_37_NO_5_S | [24] |
| 13,14-Dihydro-15-keto-PGE2 | HMDB0002776 | C04671 | C_20_H_32_O_5_ | [25] |
| 6-Keto-prostaglandin F1a | HMDB0002886 | C05961 | C_20_H_34_O_6_ | [18] |
| Leukotriene D4 | HMDB0003080 | C05951 | C_25_H_40_N_2_O_6_S | [19] |
| Hydrogen peroxide | HMDB0003125 | C00027 | H_2_O_2_ | [19] |
| Prostaglandin G2 | HMDB0003235 | C05956 | C_20_H_32_O_6_ | [25] |
| Thromboxane B2 | HMDB0003252 | C05963 | C_20_H_34_O_6_ | [26] |
| Nitric oxide | HMDB0003378 | C00533 | NO | [27] |
| 15-HETE | HMDB0003876 | C04742 | C_20_H_32_O_3_ | [18] |
| 11-Dehydro-thromboxane B2 | HMDB0004242 | C05964 | C_20_H_32_O_6_ | [25] |
| 4-Hydroxynonenal | HMDB0004362 | NA | C_9_H_16_O_2_ | [28] |
| 8-epi-PGF2alpha | HMDB0004659 | C13809 | C_20_H_40_ | [28] |
| 11-trans-LTC4 | HMDB0005095 | C02166 | C_30_H_47_N_3_O_9_S | [25] |
| Hexaldehyde | HMDB0005994 | C02373 | C_6_H_12_O | [29] |
| DL-o-Tyrosine | HMDB0006050 | NA | C_9_H_11_NO_3_ | [30] |
| 12-HETE | HMDB0006111 | C14777 | C_20_H_32_O_3_ | [23] |
| Malondialdehyde | HMDB0006112 | C19440 | C_3_H_4_O_2_ | [31] |
| 12-Hydroxyeicosapentaenoate | HMDB0010202 | NA | C_20_H_30_O_3_ | [23] |
| 5-HETE | HMDB0011134 | C04805 | C_20_H_32_O_3_ | [25] |
| 15-epi-lipoxin A4 | HMDB0012587 | NA | C_20_H_32_O_5_ | [32] |
| DL-Tryptophan | HMDB0013609 | C00525 | C_11_H_12_N_2_O_2_ | [10] |
| Iron | HMDB0015531 | C14819 | Fe | [15] |
| Zinc | HMDB0015532 | C00038 | Zn | [15] |
| Pentanal | HMDB0031206 | NA | C_5_H_10_O | [33] |
| Heptanal | HMDB0031475 | C14390 | C_7_H_14_O | [34] |
| Ammonium | HMDB0041827 | C01342 | H_4_N | [35] |
| Nonanal | HMDB0059835 | NA | C_9_H_18_O | [34] |
| Glutathionate | HMDB0062697 | C00051 | C_10_H_17_N_3_O_6_S | [36] |
| LysoPA(22:4) | HMDB0114752 | NA | C_25_H_43_O_7_P | [37] |
| 12,13-dihydroxy-9-octadecenoic acid | NA | NA | C_18_H_34_O_4_ | [38] |
| Eoxin C4 | NA | NA | C_30_H_47_N_3_O_9_S | [18] |
| Eoxin D4 | NA | NA | C_25_H_40_N_2_O_6_S | [18] |

**References**

[1] Bruderer T, Gaisl T, Gaugg MT, et al. On-line analysis of exhaled breath focus review. Chem Rev, 2019, 119: 10803-10828

[2] Schymanski EL, Jeon J, Gulde R, et al. Identifying small molecules via high resolution mass spectrometry: Communicating confidence. Environ Sci Technol, 2014, 48: 2097-2098

[3] de Laurentiis G, Paris D, Melck D, et al. Separating smoking-related diseases using nmr-based metabolomics of exhaled breath condensate. J Proteome Res, 2013, 12: 1502-1511

[4] Csoma Z, Huszár E, Vizi E, et al. Adenosine level in exhaled breath increases during exercise-induced bronchoconstriction. Eur Respir J, 2005, 25: 873-878

[5] Tomasiak-Lozowska MM, Zietkowski Z, Przeslaw K, et al. Inflammatory markers and acid-base equilibrium in exhaled breath condensate of stable and unstable asthma patients. Int Arch Allergy Immunol, 2012, 159: 121-129

[6] Baker EH, Clark N, Brennan AL, et al. Hyperglycemia and cystic fibrosis alter respiratory fluid glucose concentrations estimated by breath condensate analysis. J Appl Physiol (1985), 2007, 102: 1969-1975

[7] Corradi M, Folesani G, Andreoli R, et al. Aldehydes and glutathione in exhaled breath condensate of children with asthma exacerbation. Am J Respir Crit Care Med, 2003, 167: 395-399

[8] Greenwald R, Fitzpatrick AM, Gaston B, et al. Breath formate is a marker of airway s-nitrosothiol depletion in severe asthma. PLoS One, 2010, 5: e11919

[9] Sachs-Olsen C, Sanak M, Lang AM, et al. Eoxins: A new inflammatory pathway in childhood asthma. J Allergy Clin Immunol, 2010, 126: 859-867.e859

[10] van der Sluijs KF, van de Pol MA, Kulik W, et al. Systemic tryptophan and kynurenine catabolite levels relate to severity of rhinovirus-induced asthma exacerbation: A prospective study with a parallel-group design. Thorax, 2013, 68: 1122-1130

[11] Vlasic V, Trifunovic J, Cepelak I, et al. Urates in exhaled breath condensate of children with obstructive sleep apnea. Biochem Med (Zagreb), 2011, 21: 139-144

[12] Esther CRJ, Boysen G, Olsen BM, et al. Mass spectrometric analysis of biomarkers and dilution markers in exhaled breath condensate reveals elevated purines in asthma and cystic fibrosis. Am J Physiol Lung Cell Mol Physiol, 2009, 296: 987-993

[13] Niimi A, Nguyen LT, Usmani O, et al. Reduced ph and chloride levels in exhaled breath condensate of patients with chronic cough. Thorax, 2004, 59: 608-612

[14] Griese M, Noss J, Schramel P. Elemental and ion composition of exhaled air condensate in cystic fibrosis. J Cyst Fibros, 2003, 2: 136-142

[15] Corradi M, Acampa O, Goldoni M, et al. Metallic elements in exhaled breath condensate of patients with interstitial lung diseases. J Breath Res, 2009, 3: 046003

[16] Montuschi P, Paris D, Melck D, et al. Nmr spectroscopy metabolomic profiling of exhaled breath condensate in patients with stable and unstable cystic fibrosis. Thorax, 2012, 67: 222-228

[17] Peroni DG, Bodini A, Corradi M, et al. Markers of oxidative stress are increased in exhaled breath condensates of children with atopic dermatitis. Br J Dermatol, 2012, 166: 839-843

[18] Sanak M, Gielicz A, Bochenek G, et al. Targeted eicosanoid lipidomics of exhaled breath condensate provide a distinct pattern in the aspirin-intolerant asthma phenotype. J Allergy Clin Immunol, 2011, 127: 1141-1147.e1142

[19] Antczak A, Ciebiada M, Pietras T, et al. Exhaled eicosanoids and biomarkers of oxidative stress in exacerbation of chronic obstructive pulmonary disease. Arch Med Sci, 2012, 8: 277-285

[20] Ono E, Mita H, Taniguchi M, et al. Increase in inflammatory mediator concentrations in exhaled breath condensate after allergen inhalation. J Allergy Clin Immunol, 2008, 122: 768-773.e761

[21] Di Gangi IM, Pirillo P, Carraro S, et al. Online trapping and enrichment ultra performance liquid chromatography-tandem mass spectrometry method for sensitive measurement of "arginine-asymmetric dimethylarginine cycle" biomarkers in human exhaled breath condensate. Anal Chim Acta, 2012, 754: 67-74

[22] Hanazawa T, Kharitonov SA, Barnes PJ. Increased nitrotyrosine in exhaled breath condensate of patients with asthma. Am J Respir Crit Care Med, 2000, 162: 1273-1276

[23] Croasdell A, Thatcher TH, Kottmann RM, et al. Resolvins attenuate inflammation and promote resolution in cigarette smoke-exposed human macrophages. Am J Physiol Lung Cell Mol Physiol, 2015, 309: L888-901

[24] Carraro S, Corradi M, Zanconato S, et al. Exhaled breath condensate cysteinyl leukotrienes are increased in children with exercise-induced bronchoconstriction. J Allergy Clin Immunol, 2005, 115: 764-770

[25] Glowacka E, Jedynak-Wasowicz U, Sanak M, et al. Exhaled eicosanoid profiles in children with atopic asthma and healthy controls. Pediatr Pulmonol, 2013, 48: 324-335

[26] Huszár E, Szabó Z, Jakab A, et al. Comparative measurement of thromboxane a2 metabolites in exhaled breath condensate by different immunoassays. Inflamm Res, 2005, 54: 350-355

[27] Montuschi P, Martello S, Felli M, et al. Liquid chromatography/mass spectrometry analysis of exhaled leukotriene b4 in asthmatic children. Respir Res, 2005, 6: 119

[28] Syslová K, Kacer P, Kuzma M, et al. Rapid and easy method for monitoring oxidative stress markers in body fluids of patients with asbestos or silica-induced lung diseases. J Chromatogr B Analyt Technol Biomed Life Sci, 2009, 877: 2477-2486

[29] Corradi M, Rubinstein I, Andreoli R, et al. Aldehydes in exhaled breath condensate of patients with chronic obstructive pulmonary disease. Am J Respir Crit Care Med, 2003, 167: 1380-1386

[30] Syslová K, Kačer P, Kuzma M, et al. Lc-esi-ms/ms method for oxidative stress multimarker screening in the exhaled breath condensate of asbestosis/silicosis patients. J Breath Res, 2010, 4: 017104

[31] Antus B, Harnasi G, Drozdovszky O, et al. Monitoring oxidative stress during chronic obstructive pulmonary disease exacerbations using malondialdehyde. Respirology, 2014, 19: 74-79

[32] Kazani S, Planaguma A, Ono E, et al. Exhaled breath condensate eicosanoid levels associate with asthma and its severity. J Allergy Clin Immunol, 2013, 132: 547-553

[33] Li Y, Xu H. Development of a novel graphene/polyaniline electrodeposited coating for on-line in-tube solid phase microextraction of aldehydes in human exhaled breath condensate. J Chromatogr A, 2015, 1395: 23-31

[34] Xu H, Wei Y, Zhu L, et al. Bifunctional magnetic nanoparticles for analysis of aldehyde metabolites in exhaled breath of lung cancer patients. J Chromatogr A, 2014, 1324: 29-35

[35] MacGregor G, Ellis S, Andrews J, et al. Breath condensate ammonium is lower in children with chronic asthma. Eur Respir J, 2005, 26: 271-276

[36] Dut R, Dizdar EA, Birben E, et al. Oxidative stress and its determinants in the airways of children with asthma. Allergy, 2008, 63: 1605-1609

[37] Montesi SB, Mathai SK, Brenner LN, et al. Docosatetraenoyl lpa is elevated in exhaled breath condensate in idiopathic pulmonary fibrosis. BMC Pulm Med, 2014, 14: 5

[38] Nording ML, Yang J, Hegedus CM, et al. Endogenous levels of five fatty acid metabolites in exhaled breath condensate to monitor asthma by high-performance liquid chromatography: Electrospray tandem mass spectrometry. IEEE Sens J, 2010, 10: 123-130
